# Nasal Birth Trauma: A Review of Appropriate Treatment

**DOI:** 10.1155/2010/752974

**Published:** 2010-12-19

**Authors:** E. C. Cashman, Terence Farrell, M. Shandilya

**Affiliations:** ^1^Department of Otolaryngology, Waterford Regional Hospital, Waterford, Ireland; ^2^Department of Otolaryngology, Head and Neck Surgery, St. James's Hospital, Dublin, Ireland

## Abstract

The aetiology of nasal deformity has frequently included birth trauma. There is no consensus in the literature as to whether nasal surgery, in the form of closed reduction, is indicated in neonates. The majority of studies in the literature that advocate intervention have inadequate followup periods and there is a paucity of evidence for the adverse effects of conservative management. This case highlights the therapeutic dilemma posed by such nasal injuries in the neonate and, to the best of the authors' knowledge, at the time of writing, represents the earliest reported case in the literature of nasal deformity in the neonate. The term nasal deformity is used to denote deformity of the nasal pyramid, soft tissue, and septum. Three main aspects of neonatal nasal deformity are addressed including, firstly, if nasal deformity at birth needs to be addressed, secondly, if left unaltered, what the long-term effects are and, finally, if intervention alters the normal course of midfacial development.

## 1. Introduction

Nasal deformity occurs more frequently during childhood although it occurs at any age due to trauma. Birth trauma was first reported by Metzenbaum in 1929 as being a causative factor in nasal septal deviation [1]. Traditionally, surgery for nasal deformity in children is not usually preformed until an age close to 16 years. To the best of the authors' knowledge, at the time of writing, this case represents the earliest reported case of nasal deformity in the neonatal setting.

## 2. Case Report

**Figure 1:**
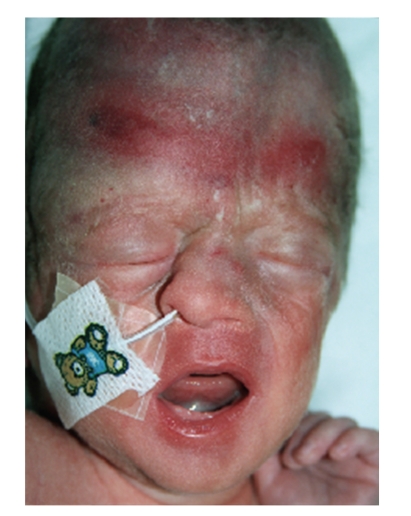
At 2 hours after delivery.

**Figure 2:**
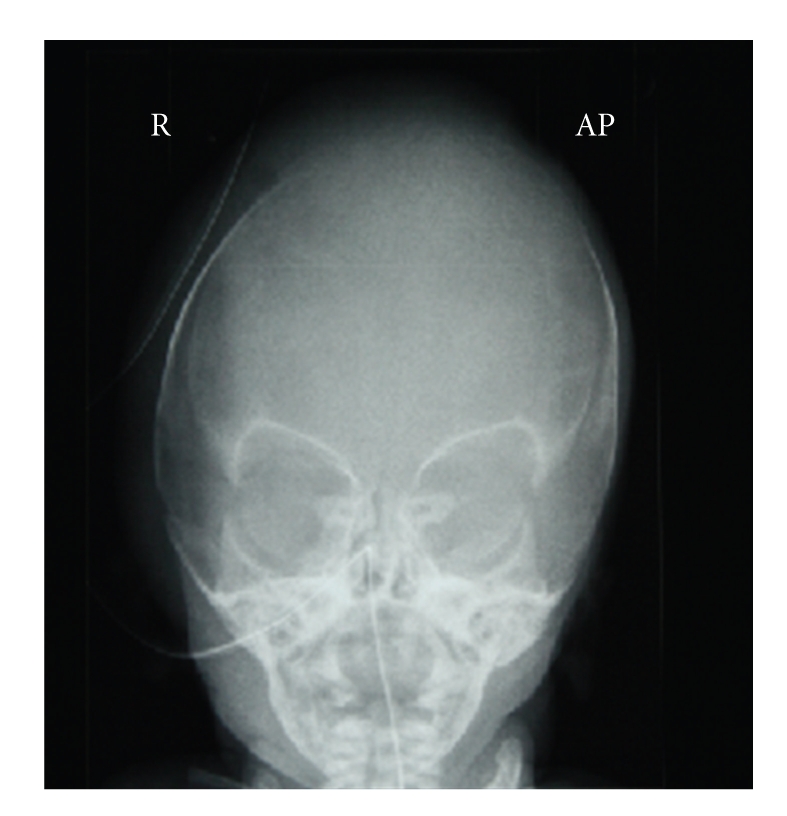
X-Ray demonstrating deviation nasal septum to right.

**Figure 3:**
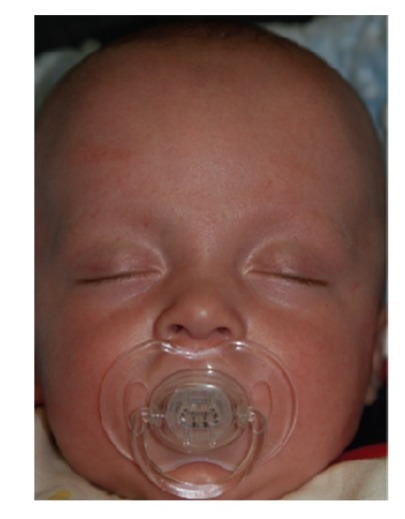
At six weeks.

This is the case of a 2-hour-old neonate delivered transvaginally at 38 weeks. His mother was a primigravida, with no comorbidities, and had an uneventful pregnancy. The delivery was forceps assisted. The Apgar score was 9 at 1 min and 10 at 5 minutes. On examination he had a markedly deviated nasal septum (Figure 1), and an X-ray of nasal bones (Figure 2) demonstrated a deviated nasal septum and compression of the midface. The patient demonstrated no signs of respiratory distress so closed reduction was not preformed. A decision was made to follow the patient up in the otolaryngology outpatient department at six weeks (Figure 3). 

## 3. Discussion

Nasal deformity in the newborn is said to arise due to various intrauterine and transnatal pressures operating on the fetus. In 1963, Klaff reported 12 cases of neonatal deformity and went on to discuss causative factors and methods of treatment [2]. Gray investigated septal deformities in 2,380 infants at birth and found anterior cartilage deformity in 4% [3]. Hartikainen detected a 1.9% incidence of anterior septal dislocation in neonates and proposed that the majority of dislocations occurred during intrauterine life [4]. More recently Bhattacharsee et al. noted an incidence of 14.5% [5].

Nasal fracture is the most common facial fracture, and the prevalence of nasal septal deformity and its relationship with the different types and difficulty of delivery have been assessed in a variety of studies. Saim and said, in a randomised group of newborns found 21.8% had nasal deformities [6]. They concluded that there was no significant difference in the prevalence of neonatal nasal deformity in the different types of delivery. In addition, they noted that there was no significant increase in the prevalence of nasal deformity with increasing difficulty of the delivery. In contrast, Podshin et al. found an incidence of 0.93% of anterior nasal septal cartilaginous dislocation in neonates in their series [7]. They noted definite correlation between the type of delivery and the nasal deformity. Uyger noted significant correlation between pregnancy, delivery period, the way of delivery, and the incidence of septal deviation and columella dislocation and concluded from his study that a careful rhinologic examination should be carried out in the newborns that have prolonged delivery, increased head circumference, and vaginal delivery [8].

 There are two basic types of septal deformity, anterior nasal deformity or combined nasal deformity. They may occur independently of one another or together depending on the different types of pressure on the foetus during pregnancy or parturition. Septal dislocation in many cases returns to normal position within a few days but gross deviation, as illustrated by our case, may go on to give rise to physiological, anatomical, cosmetic and psychological dysfunction. Many different and often complex methods of classifying nasal septal fracture have been proposed. Stranc and Robertson found that lateral forces account for most nasal fractures [9]. 

The external nose is a triangular pyramid composed of cartilaginous and osseous structures that support the skin, musculature, mucosa, nerves, and vascular structures. A child's nose differs from an adult in several ways. Specifically, the underdeveloped nose has less frontal projection, is largely composed of cartilage, and possesses several growth centres. The cartilaginous septum is the dominant growth centre, and loss of septal cartilage at different ages leads to different facial syndromes including nose, maxilla, and orbita. The septal cartilage in children demonstrates thinner fracture-prone areas next to thicker growth zones. As a result, paediatric nasal trauma often presents a unique diagnostic and therapeutic dilemma and traditionally the approach to deviated nasal septum in children has been largely cautious because of the potential adverse and unpredictable effects of growth spurts on midface development. Since the child's nose is more cartilaginous than its adult counterpart, it is easily compressible and absorbs little energy from any force as it passes across the face. The resultant oedema spreads over the face and tends to disguise the extent of the nasal involvement in the paediatric age group. 

In older children, the optimal timing and the extent of surgical intervention in nasal surgery is controversial, and traditionally surgery for nasal deformity is not preformed until an age close to 16 years. However, there has been a shift in opinion recently with several authors claiming a paucity of evidence for the adverse effects of conservative surgery [10, 11]. Research has demonstrated that surgical intervention limited to specific areas of the bony and cartilaginous nasal framework is less likely to impact on natural growth patterns. The situation is further compounded by the absence of any internationally recognised standardised method of assessing and quantifying midfacial growth. Studies that have advocated surgery on the paediatric nose have not employed adequate followup periods [12]. Tasca and compadretti reviewed 49 children who underwent treatment for septal dislocation within 48 hours after birth and re-examined the group at a mean age of 13.2 years [13]. They noted satisfactory anatomic and functional findings for all children affected by septal dislocation and concluded that given the simplicity and safety of the reduction manoeuvre, that early intervention and treatment, in the form of immediate reduction, was vital. 

Two key growth spurts are known to occur, the first during the first two postnatal years and the second during puberty. Traditionally it has been held that surgical intervention between these two periods may induce a growth disturbance. However, even after the final growth spurt, further growth may occur up to the age of 25, and thus postsurgical distortion is still an issue in the adult setting. Sooknundun et al. argue that neonatal nasal deformity if left alone continues to persist and may even be accompanied by symptoms such as upper respiratory tract infections, cough, earache, mouth breathing, and at times feeding difficulties. In their series, long-term followup of children who underwent closed reduction at birth, revealed no adverse effects such as nasofacial disproportion or retardation of facial growth [14]. Although nasal surgeons have advocated immediate repair of nasal deformity resulting from birth trauma, correction is only indicated in the case of nasal obstruction, as newborns are obligate nasal breathers [15, 16].

## 4. Conclusion

There is no consensus in the literature as to whether or not surgical intervention in the form of closed reduction is indicated in the neonatal period and cases such as ours prove a therapeutic dilemma. In addition, if surgery is indicated in the paediatric group the optimal timing for intervention remains ill-defined. More objective assessment and analysis is required with adequate followup and the exact sequelae of surgical manipulation in the neonatal setting have yet to be established. This case demonstrates that the need for reduction may be overemphasised and is not universal. Long-term followup and additional studies comparing intervention versus observation for nasal deformities of similar degrees of severity are needed.
